# MALDI Mass Spectral Imaging of Bile Acids Observed as Deprotonated Molecules and Proton-Bound Dimers from Mouse Liver Sections

**DOI:** 10.1007/s13361-017-1886-6

**Published:** 2018-02-07

**Authors:** Ignacy Rzagalinski, Nadine Hainz, Carola Meier, Thomas Tschernig, Dietrich A. Volmer

**Affiliations:** 1 Institute of Bioanalytical Chemistry, Saarland University, 66123 Saarbrücken, Germany; 2 Institute of Anatomy and Cell Biology, Saarland University, 66421 Homburg, Germany; 3 Department of Chemistry, Humboldt University of Berlin, 12489 Berlin, Germany

**Keywords:** Mass spectrometry imaging, MALDI, FTICR, Bile acids, Taurocholic acid, Taurine, Proton-bound dimers, Adducts

## Abstract

Bile acids (BAs) play two vital roles in living organisms, as they are involved in (1) the secretion of cholesterol from liver, and (2) the lipid digestion/absorption in the intestine. Abnormal bile acid synthesis or secretion can lead to severe liver disorders. Even though there is extensive literature on the mass spectrometric determination of BAs in biofluids and tissue homogenates, there are no reports on the spatial distribution in the biliary network of the liver. Here, we demonstrate the application of high mass resolution/mass accuracy matrix-assisted laser desorption/ionization (MALDI)-Fourier-transform ion cyclotron resonance (FTICR) to MS imaging (MSI) of BAs at high spatial resolutions (pixel size, 25 μm). The results show chemical heterogeneity of the mouse liver sections with a number of branching biliary and blood ducts. In addition to ion signals from deprotonation of the BA molecules, MALDI-MSI generated several further intense signals at larger *m/z* for the BAs. These signals were spatially co-localized with the deprotonated molecules and easily misinterpreted as additional products of BA biotransformations. In-depth analysis of accurate mass shifts and additional electrospray ionization and MALDI-FTICR experiments, however, confirmed them as proton-bound dimers. Interestingly, dimers of bile acids, but also unusual mixed dimers of different taurine-conjugated bile acids and free taurine, were identified. Since formation of these complexes will negatively influence signal intensities of the desired [M – H]^-^ ions and significantly complicate mass spectral interpretations, two simple broadband techniques were proposed for non-selective dissociation of dimers that lead to increased signals for the deprotonated BAs.

**Electronic supplementary material:**

The online version of this article (10.1007/s13361-017-1886-6) contains supplementary material, which is available to authorized users.

**Graphical Abstract:**
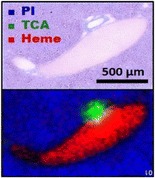
ᅟ

## Introduction

Bile acids (BAs) are an integral part of the digestive system of living organisms, where they play two essential roles: firstly, they are synthesized from cholesterol in the liver hepatocytes and this process maintains cholesterol homeostasis. Secondly, bile acids in their conjugated forms (“bile salts”) participate in digestion and absorption of lipids and lipid soluble compounds (e.g., vitamins) in the intestine [1]. Interestingly, difference of bile acids composition exist between vertebrates, which makes this an interesting biochemical trait not only from an evolutionary biology point of view, but also from a biomedical perspective [2]. For example, recent reports have shown a link between characteristic BA composition in mice and their resistance to high fat diet-induced obesity and diabetes [3–5]. In contrast to these beneficial effects, some bile acids (e.g., litocholic acid) are considered toxic endobiotics [6]. Moreover, abnormalities in BA synthesis (which is tightly controlled by 17 different enzymes) as well as secretion processes can lead to development of serious diseases, including bile acid (inborn) synthesis disorder and primary biliary cholangitis [7–9]. Furthermore, accumulation of BAs in undesirable organs/tissues causes oxidative/nitrosative stress, damages of DNA and cell apoptosis [10–12].

**Figure 1:**
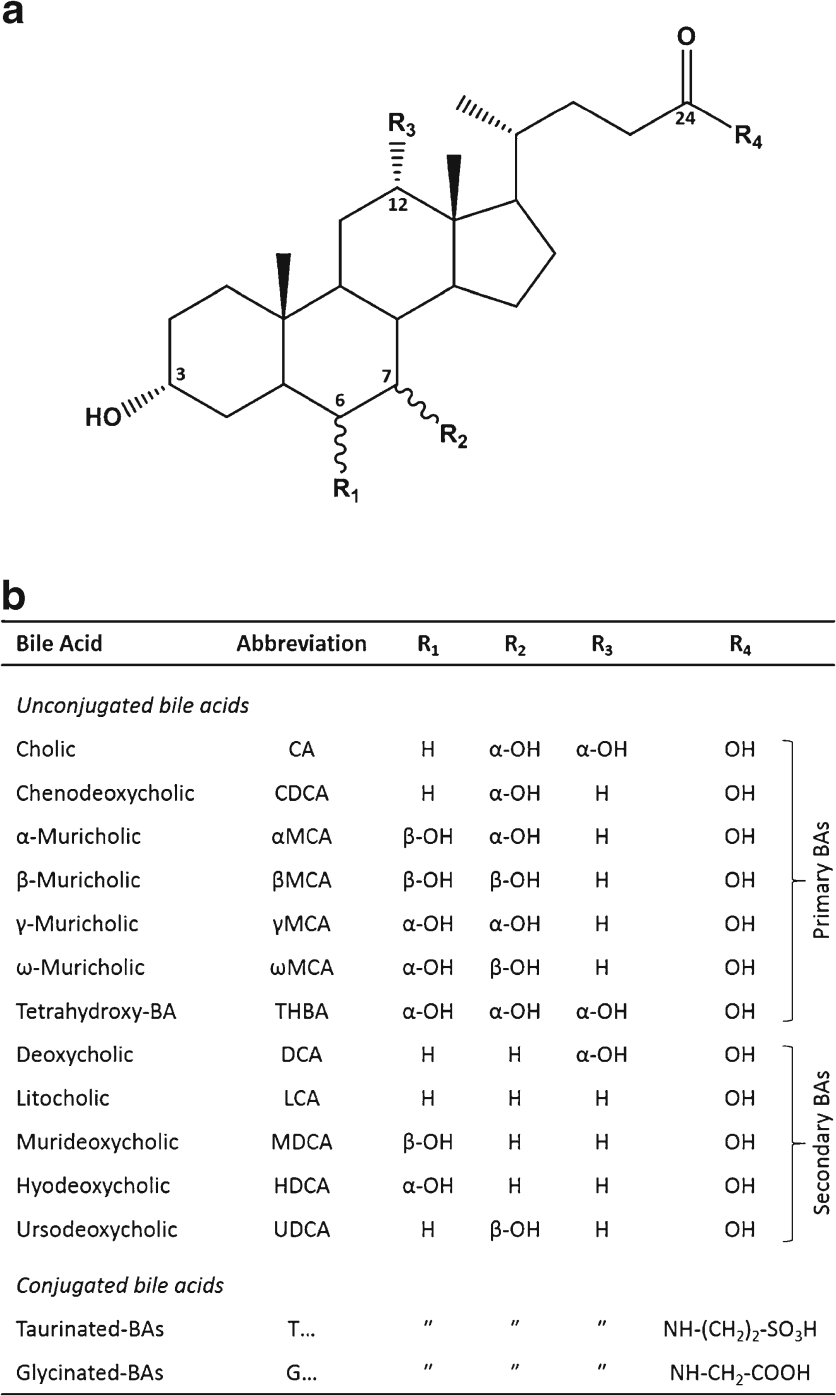
**(a)** Typical four-ring steroid structures of 24-carbon bile acids, including differences in number and positions of hydroxyl groups (R_1_-R_3_) as well as N-amide conjugation (R_4_). **(b)** Most common non-conjugated bile acids present in humans and rodents, with the different positions of hydroxyl groups as well as two major conjugation types

The complex biosyntheses of primary bile acids in liver hepatocytes results in large structural diversity of endogenous metabolites. In addition, secondary bile acids are formed in the colon as a result of bacterial action, and some of them re-enter hepatic circulation (enterohepatic recirculation) [13]. In mammals, bile composition is almost exclusively limited to compounds consisting of 24 carbon atoms; that is, acidic steroids with typical four-ring steroid core and C-5 side chain (Figure 1a). They differ in the number and position of hydroxyl groups attached to the rings and also include epimers, for example, muricholic acid (characteristic for mice) with four different combinations of two hydroxyl groups attached to C-6 and C-7 positions. In addition, unconjugated BAs undergo further biotransformation routes, from which the most important is N-acyl amidation with taurine or glycine at C-24. Moreover, conjugations at C-3, C-6, or C-7 include sulfation, glucuronidation, glucosidation, and N-acetylglucosaminidation [14, 15]. The nomenclature of bile acids was developed by Hoffman et al. [16] (Figure 1 summarizes structures and abbreviations of the most common human and mouse bile acids). There is a large group of intermediate products of 24 carbon bile acids containing a keto group at C-3 (oxo-BAs) and/or unsaturated double bonds at C-4 or C-6. The latter were used as potential biomarkers of pathological change in severe liver diseases [7, 10].

All these processes combined with recent findings presenting bile acids as pleiotropic signaling molecules [17, 18] require powerful analytical assays to fully decipher the complete molecular signatures of BAs in different biological matrices and link them to the pathology of the corresponding disease. As a result, MS-based techniques have been the primary analytical tools for bile acid characterization over the last few decades. Initially, fast-atom bombardment (FAB) was often implemented, delivering mostly deprotonated molecules for BAs in negative ion mode. FAB was frequently used with double focusing magnetic sector instruments, which allowed high-energy collisions (>1 keV) and subsequent dissociation of the steroid backbone via charge-remote fragmentation (CRF) [19, 20]. Of note, conjugation through N-acyl amidation of the side chain has two practical implications for BAs. Firstly, it increases acidity (e.g., p*K*_a_ of TCA ~1.5 versus CA ~4.5) and thus improves ionization efficiency. Secondly, it enlarges cross-sections for CRF, resulting in intense and informative MS/MS spectra. This phenomenon was used to improve FAB-MS of bile acids after derivatization with taurine or other aminosulphonates [21, 22]. Other ionization techniques were also used, including thermospray [23, 24], atmospheric pressure chemical ionization [25–28] – both coupled to liquid chromatography – and electron ionization after derivatization/separation by gas chromatography [29, 30]. Today, matrix-assisted laser desorption/ionization (MALDI) and electrospray ionization (ESI) dominate analysis of BAs [31]. Particularly LC-ESI-MS/MS is the method of choice for analysis from different biological samples such as biofluids (urine, bile, plasma/serum) [32–34] and liver tissue homogenates [35, 36]. Efficient LC separation is vital, as LC-MS/MS instruments usually only provide low energy collision induced dissociation (CID) (0–100 eV), with less structure-informative spectra as high-energy CID [19]. In contrast to ESI, MALDI has not been extensively applied to analysis of BAs, except for the quantification of bile acids in urine and plasma [37, 38] and for MALDI imaging of taurocholic acid in a murine model of polycystic kidney disease [39]. Mass spectrometry imaging (MSI), however, which combines detailed molecular characterization with spatial distribution measurements, has not yet been applied for determining spatial distribution of BAs in mouse liver biliary networks, which is the main purpose of the present work. In addition, we were concerned with unusual adducts observed during MALDI-MSI analysis of bile acids.

Adduct formation is common in mass spectrometry and adducts often seriously complicate mass spectral interpretation [40]; sometimes they enhance detection when deliberately formed (e.g., lithium adducts of phospholipids [41] or silver adducts of cholesterol [42]). Interestingly, gas-phase ESI or MALDI adducts are often stable in positive ion mode. Anion adducts, however, often do not survive the MALDI process, which is attributed to the larger amount of energy transferred to the desorbed molecules upon ionization [43, 44]. A particular type of ionic cluster is the proton-bound dimer (PBD), which consists of the same (=homodimer) or different (=heterodimer) species (ions or neutrals). Their weak hydrogen bonding was used as principle of the *kinetic method*, which was developed by Cook and coworkers [45–47] and later refined, e.g., by the Fenselau group [48, 49]. In this method, ESI combined with CID of *m/z*-selected PBD is used to determine relative proton affinities (PA) of ions contributing to the isolated cluster. Bile acids were investigated by this method and exhibited different MS/MS spectra, depending on the PA of the contributing ions [50, 51].

The present work reports on proton-bound dimers formed during MALDI and the extensive presence of these clusters from endogenous tissue metabolites in MSI. Specifically, we describe application of high resolution FTICR-MS at high MALDI-MSI spatial resolving powers (25 μm) for bile acids in the biliary network of mouse liver sections and demonstrate identification of taurine-conjugated bile acids directly from tissue sections and formation of proton-bound dimers of different bile acids and taurine.

## Experimental

### Materials and Reagents

Taurine (99%), 9-aminoacridine (9-AA, 99.5%), ethanol (HPLC grade) and standard microscopic glass slides were purchased from Sigma-Aldrich (Steinheim, Germany). Taurocholic acid (sodium salt, 95%) was from Biomol GmbH (Hamburg, Germany), and potassium chloride (99.5%) from Gruessing GmbH (Filsum, Germany). Purified water was generated by a Millipore (Bedford, MA, USA) purification system.

### Animals and Tissue Preparation

All animal experiments were performed in accordance with international regulations and permission from the local research ethics committee (Saarland Government TVV 27/2014). Female C57BL/6 mice (12-wk) were purchased (Charles River, Sulzbach, Germany). The animals were anesthetized with xylazine/ketamine and organs were dissected immediately after sacrifice, snap-frozen in liquid N_2_, and stored at –80 °C until the sample preparation process. The longitudinal mouse liver sections were prepared at 12 μm thicknesses using a Reichert Jung 2800 Frigocut cryostat microtome (Leica Microsystems, Wetzlar, Germany), thaw-mounted onto the plain microscope glass slides, and dried for 30 min in a vacuum desiccator. The tissues were then stored at –80 °C prior to mass spectrometry imaging experiment.

### MALDI Matrix Deposition and Evaluation of its Quality

The MALDI matrix 9-aminoacridine was chosen in this study because of its ability to efficiently ionize acidic compounds in negative ion mode, including bile acids [37–39]. A solution of 9-AA (5 mg/mL) was freshly prepared in ethanol/water (70/30 [v/v]) prior to deposition. Matrix was applied on top of the tissue sections using a home-built automatic sprayer, based on the Probot micro fraction collector (Thermo Scientific, Germering, Germany) and MicroIonSpray nebulizing nozzle (Sciex, Concord, ON, Canada). Fused silica spray capillaries (Molex Polymicro Technologies, USA) of several different internal diameters were tested with respect to homogeneity of matrix layers, crystal sizes, and resistance to clogging during the longer spraying process. Eventually, the capillaries of 200 μm internal diameter were chosen as they provided optimum performance. The nozzle height was 44 mm, distance between the lines was 3 mm, and the movement speed was 199.25 mm/min along the y-axis in a meandering pattern. For final imaging experiments, seven layers of 9-AA were sprayed with increasing flow rate in the following pattern: two layers at 20 μL/min and five layers at 40 μL/min. From these experiments, the estimated amount of matrix added to the tissue (=matrix density) was calculated at 0.0201 mg/mm^2^. Eventually, in-depth visual inspection of the obtained 9-AA layers was carried out using optical and scanning electron (SEM) microscope images, which revealed high homogeneity and reproducibility as well as crystal sizes down to 1 μm (Figure S1, Supplementary Material).

### Mass Spectrometry and Data Analysis

MALDI imaging, MALDI and ESI experiments were performed in negative ion mode on a Bruker (Bremen, Germany) 7 Tesla Solarix FTICR mass spectrometer, equipped with a dual ESI/MALDI ion source and Smartbeam II Nd:YAG (355 nm) laser. MALDI imaging data were collected from *m/z* 50 to 2000 with a transient length of ~0.5 s and resolving power (FWHM) of ~90,000 at *m/z* 400. Internal mass calibration was performed using a series of peaks originating from MALDI matrix and endogenous compounds. Two different MSI pixel size settings were used: 70 μm for low spatial resolution imaging of the whole liver section and 25 μm for the high spatial resolution imaging of selected sub-regions of the adjacent tissue section. This particular approach was chosen because of analysis time considerations. The run time for the whole liver section at the larger raster step size of 70 μm was ca. 8 h (approximately 22,000 single points). The same whole section imaged at 25 μm would have required ~189,000 points, with an estimated total run time exceeding 63 h. Selecting the smaller regions of interest for imaging at high spatial resolution, however, allowed us to keep the single experiment analysis within a few hours. For low resolution experiments, the laser was set to the “small” spot size, laser power to 20%, number of laser shots/pixel to 100, and frequency to 1 kHz. For high spatial resolution experiments, different laser settings were tested with respect to signal intensities as well as to ablated area, assuring dense pixel deposition and avoiding overlap between neighboring laser spots (Figure S2, Supplementary Material). As a result of these optimization experiments, the optimal laser settings were set to “minimum” spot size, laser power to 10%, number of laser shots/pixel to 200, and frequency to 1 kHz. MALDI experiments with standards were carried out by using dried-droplet sample preparation and spotting onto steel MALDI target plates (Bruker). ESI was performed using direct infusion using the instrument’s syringe pump at 2 μL/min. All MS/MS experiments were performed by isolation of precursor ions in the external quadrupole (isolation window: 3–5 u) and accumulation in the hexapole for collision-induced dissociation (CID) at varying collision energies (5–55 V). Data were processed and analyzed using the Bruker Data Analysis and FlexImaging software for single mass spectra and imaging data sets, respectively. Mass spectral interpretations were conducted using the METLIN and LIPID MAPS databases to match the accurate masses of the precursor ions, and by manually interpreting MS/MS fragmentation patterns and comparing with standards. MS images were normalized to the deprotonated 9-AA signal, which was previously described as an efficient normalization routine when isotope labeled internal standards are not available [52–55]. This approach was chosen here over the more common TIC and RMS routines because both TIC and RMS caused unrealistic enhancements of ion yields in our MSI data sets (data not shown). Such artifacts, in particular for signals of high intensity in certain regions, which comply well with the histological structures of heterogeneous tissues, have previously been described by Deininger et al. [56].

### Histological Staining and Optical Microscopy

After MALDI-MSI measurements, the glass slides were gently washed with 70% ethanol to remove the matrix. The tissue sections were stained with hematoxylin and eosin (H&E) as follows: glass slides were dipped in 100% ethanol at –20 °C for 5 min, followed by distilled water for 2 min. The slides were then immersed in Ehrlich’s hematoxylin (Carl Roth, Karlsruhe, Germany) for 5 min, washed with deionized water, followed by bluing in running tap water for 10 min. Staining by eosin (Carl Roth) for 10 s was followed by a wash with deionized water. Slides were then dipped in 80% ethanol for 3 min, 90% ethanol for 6 min, and 100% ethanol for 2 × 5 min, followed by placing them in three different xylene (VWR, Darmstadt, Germany) baths for 5 min each. Finally, slides were cover slipped using mounting medium Roti-Histokitt (Carl Roth GmbH, Karlsruhe, Germany). The stained tissue sections were scanned using an Olympus dotslide optical microscope with an UPLANSAPO 40x/0.90 objective (Olympus, Tokyo, Japan).

## Results and Discussion

### On-Tissue Identification of Taurine Conjugated Bile Acids

Because of the chemical complexity of the bile acid lipid class, high resolution/high accuracy MALDI-FTICR was employed to identify the molecules directly from mouse liver sections. In the first step, interpretation of the average as well as single pixel mass spectra from the MSI experiment was performed and annotated *m/z* values (mass uncertainty, ±3 ppm) within the bile duct and gall bladder regions were interrogated using the METLIN (http://metlin.scripps.edu) and LIPID MAPS (http://www.lipidmaps.org) databases. Tentative identifications were compared with an existing study of LC-MS/MS analysis of mouse bile, plasma, and liver tissue homogenates [32–34]. In the second step, CID experiments were performed directly on-tissue (bile duct and/or gall bladder regions) and MS/MS spectra manually interpreted.

**Figure 2:**
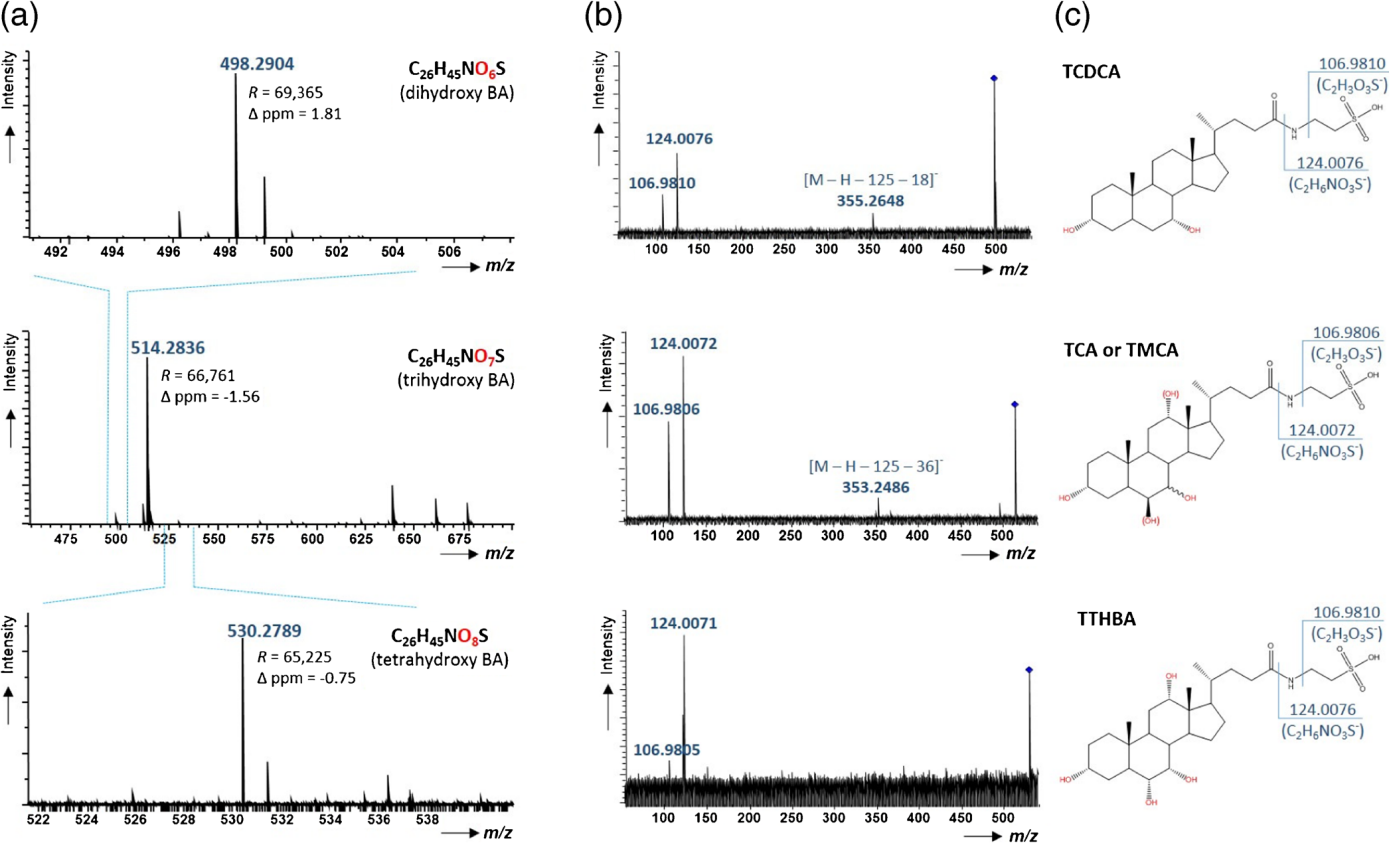
**(a)** MSI single pixel mass spectrum for the *m/z* range 450–700 (center), with two smaller expanded *m/z* range: *m/z* 492–508 (top) and *m/z* 522–540 (bottom), showing signals from deprotonated molecules of trihydroxy, dihydroxy, and tetrahydroxy bile acids, respectively. **(b)** CID mass spectra of three identified BAs, confirming taurine-conjugated side chains, obtained after isolation in the quadrupole and dissociation in the hexapole collision cell at energies of 50, 55, and 50 eV for dihydroxy, trihydroxy, and tetrahydroxy BAs, respectively. **(c)** Structures of the three identified BAs (TCA and TMCA isomers are both shown, as distinction was not possible with the present method)

A representative MALDI mass spectrum extracted from a single pixel (bile duct region) of the MSI experiment is shown in Figure 2a. The measured accurate *m/z* of the base peak at *m/z* 514.2836 (center panel) matched that of the [M – H]^-^ ion of a compound with molecular formula C_26_H_45_NO_7_S (exact mass, *m/z* 514.2844), which in turn was assigned to taurocholic acid (TCA) or one of the epimers of tauromuricholic acid (α-, β-, γ-, or ω-TMCA, Figure 1). In addition, two other important signals were found. Firstly, *m/z* 498.2904 (top panel) was assigned a molecular formula of C_26_H_45_NO_6_S and tentatively identified as taurochenodeoxycholic acid (TCDCA); secondly, *m/z* 530.2789 (bottom panel) was assigned the formula C_26_H_45_NO_8_S and tentatively identified as taurotetrahydroxycholic acid (TTHBA). The molecular formulae differed by 16 u (one oxygen), thus representing the taurine conjugates of primary bile acids containing two, three, and four hydroxyl groups at the steroid cores. CID experiments of each species (Figure 2b and c) revealed characteristic fragment ions at *m/z* 124 (C_2_H_6_NO_3_S^-^) and *m/z* 107 (C_2_H_3_O_3_S^-^), resulting from cleavages from the taurine conjugated side chain. In addition, two further meaningful fragment ions were observed, originating from neutral losses of taurine and a single water molecule (*m/z* 355.2648) or taurine and two water molecules (*m/z* 353.2486), which are characteristic diagnostic ions for dihydroxy and trihydroxy BAs [31, 57]. Of note, MS/MS-based distinction between the two individual isomers of trihydroxy bile acids (TCA and TMCA) was not possible with the implemented instrument, as only low-energy CID was available, which does not provide isomer-specific ions for the two species. However, based on quantitative data for the isomers shown in previous LC-MS/MS studies of bile acids in mouse gall bladder and liver, we assume that both isomers contribute to the ion abundance at *m/z* 514.3 [5, 33]. Potential isomer contributions will be investigated in the future by utilizing ion mobility separation after MALDI.

### High Spatial Resolution MALDI-FTICR Imaging of BAs in Mouse Liver Sections

Even though liver is often considered to be a homogenous tissue in MSI studies, it is in fact a highly vascularized organ [58]. The relatively homogenous parenchymal tissue consisting of the hepatocytes is highly permeated by branching biliary and blood vessels. Bile acids are synthesized in the hepatic lobules of liver parenchyma and, following conjugation with taurine (mice) or taurine and glycine (humans), they are secreted to the gall bladder through a branching network of bile canaliculi, canals of Hering, and different size bile ducts, which together create the characteristic biliary tree [58–60]. Here, we used MALDI-MSI to investigate molecular profiles and branching patterns of the mouse intrahepatic biliary tree at high lateral resolutions (pixel size, 25 μm). In addition to the single ion images of different bile acids, we also provide molecular differentiation between bile ducts, blood vessels and hepatic parenchymal tissue from a single tissue section, based on the RGB ion images of three different compounds serving as the molecular surrogate markers of the three regions.

**Figure 3:**
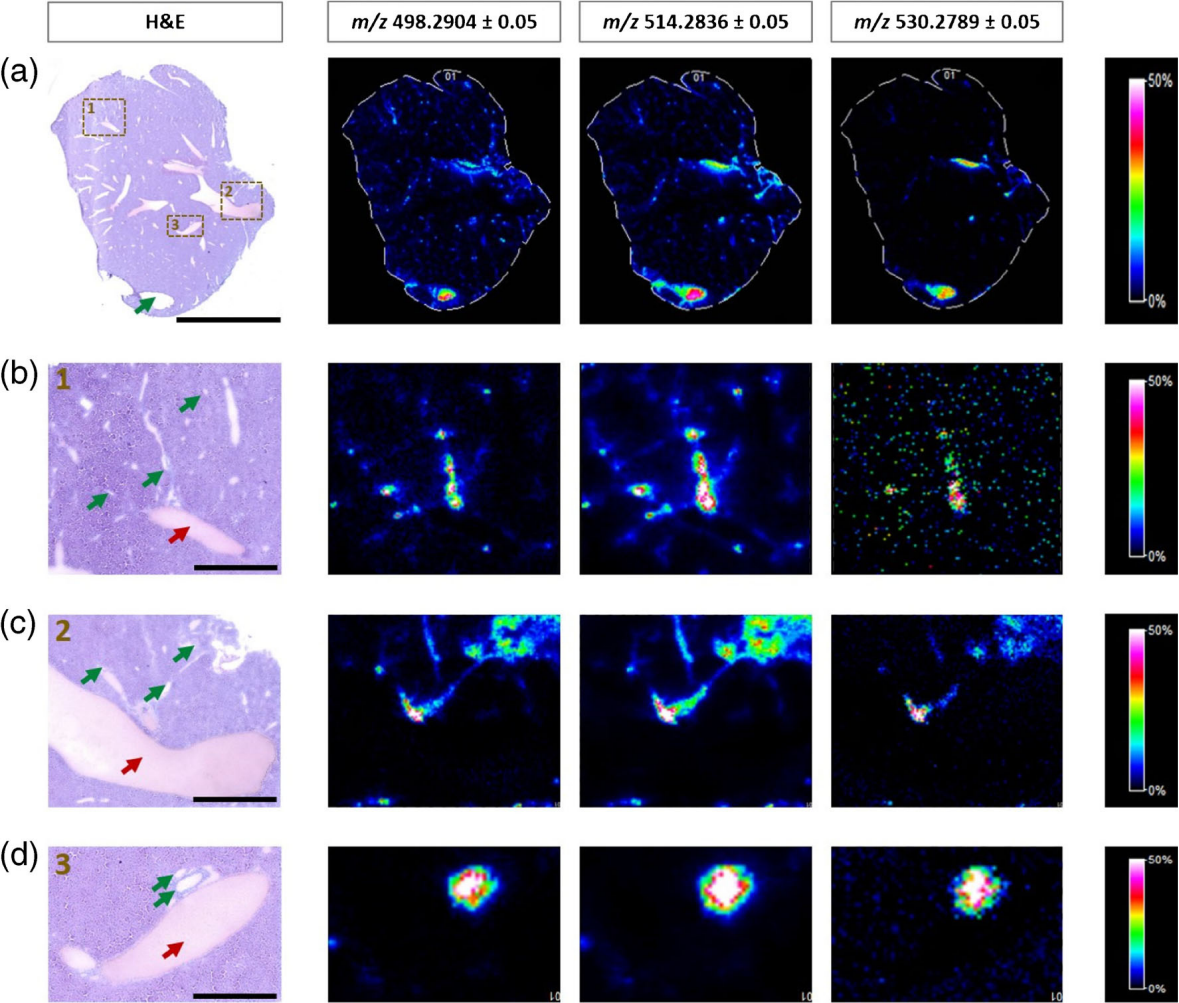
MS ion images representing spatial distributions of the identified taurine-conjugated bile acids at *m/z* 498.2904 ± 0.05 (TCDCA), *m/z* 514.2836 ± 0.05 (TCA/TMCA), and *m/z* 530.2789 ± 0.05 (TTHBA) for the whole section of mouse liver at low spatial resolution (pixel size, 70 μm) **(a)**, and in smaller sub-regions of an adjacent tissue section at high spatial resolution (pixel size, 25 μm) **(b**–**d)**. All ion images were normalized to the 9-AA matrix signal. The green arrows indicate gall bladder **(a)** or bile vessels **(b**–**d)**, whereas the red arrows show blood vessels. Scale bars: 5 mm **(a)**, 1000 μm **(b**, **c),** and 500 μm **(d)**

The spatial distributions of the three taurine-conjugated bile acids discussed above are shown in Figure 3. All ion images are created based on the signals from the deprotonated molecules: TCDCA at *m/z* 498.2904 ± 0.05, TCA/TMCA at *m/z* 514.2836 ± 0.05, and TTHBA at *m/z* 530.2789 ± 0.05. The raster width was 70 μm for whole tissue sections (Figure 3a) and 25 μm for selected smaller sub-regions of adjacent sections (Figure 3b–d). All three bile acids were clearly co-localized, correlating well with the anatomical features of bile ducts revealed by the H&E-stained images, and clearly showing the branching network of the biliary tree in the high spatial resolution images (Figure 3b). From a comparison of the relative signal intensities (normalized to 9-AA matrix signal), we determined TCA/TMCA as the most abundant investigated component of bile, and TTHBA as lowest abundant molecule. In addition, the highest intensity of all three BAs was found in the gall bladder (green arrow on the H&E image, Figure 3), which serves as storage reservoir for a concentrated bile [59, 61]. These findings are in good agreement with previously published quantitative LC-MS/MS reports [35, 36].

**Figure 4:**
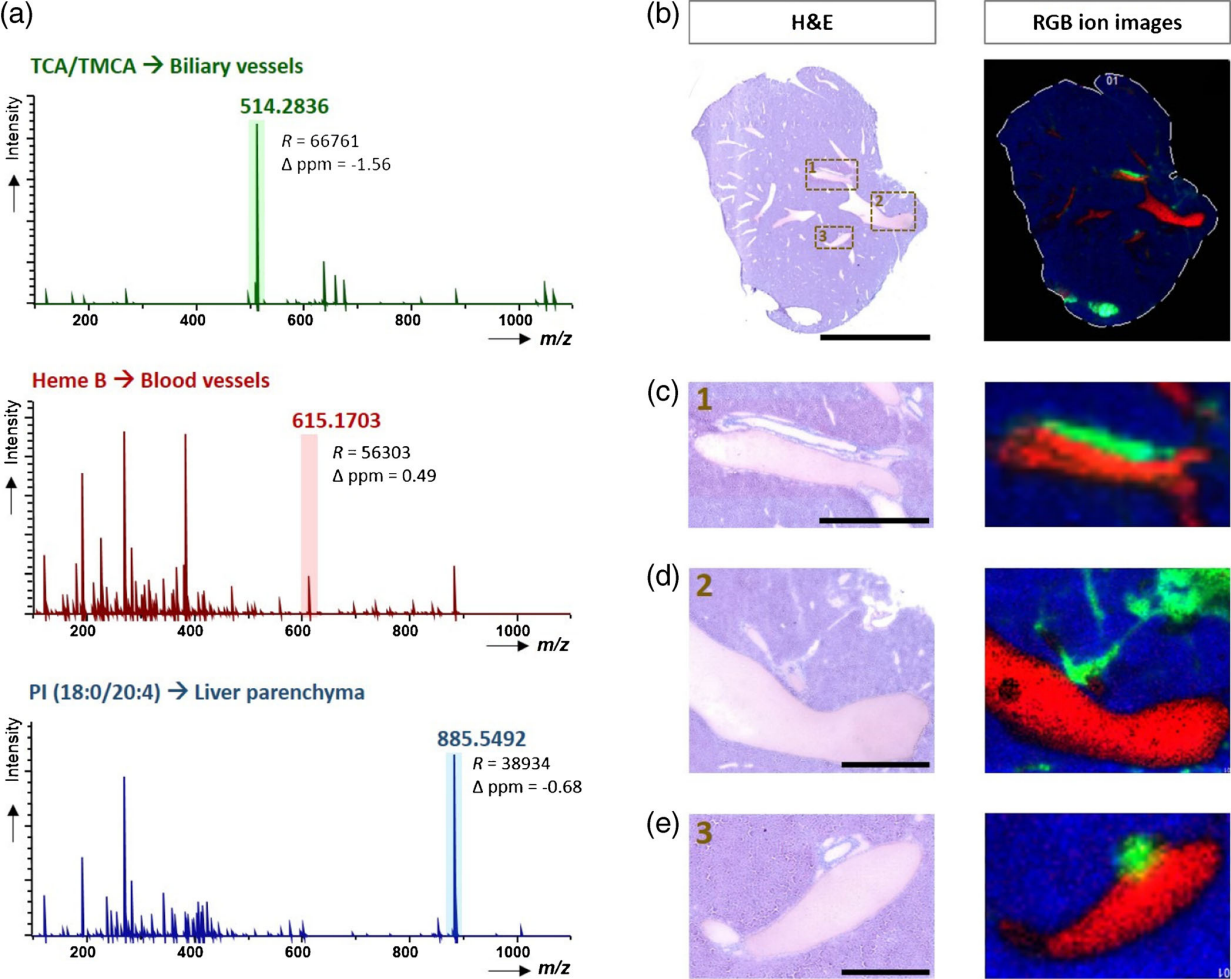
MALDI-MSI single pixel mass spectra from three regions of liver tissue section with different microstructures and chemical compositions: biliary tree (bile duct), blood vessel, and liver parenchyma. The *m/z* values used for RGB MS images are highlighted in the spectra **(a)**. RGB ion images representing three different compounds, showing clear distinction between anatomical features (as shown by the H&E stained images) across the whole tissue section (pixel size, 70 μm) **(b)**. Expansion of the smaller sub-region **(c)** as well as smaller sub-regions from an adjacent section (pixel size, 25 μm) **(d**, **e)**. Scale bars: 5 mm **(b)**, 1000 μm **(c**, **d),** and 500 μm **(e)**

In addition to determining spatial distribution of selected bile acids in liver sections, the MALDI-MSI method also enabled us to perform molecular fingerprinting of mouse liver microstructures. As seen in Figure 4a, three different regions can be distinguished based on the mass spectra collected from these areas: bile ducts, blood vessels, and liver parenchymal tissue. Furthermore, distinct molecular histology images can be readily obtained by selecting three different deprotonated molecules: *m/z* 514.2836 ± 0.05 (TCA/TMCA), 615.1703 ± 0.05 (heme B), and 885.5492 ± 0.05 (PI (18:0/20:4)), serving as surrogate molecular markers of the above-mentioned regions. Using these marker ions, Figure 4b, c illustrate RGB ion images of the whole section and the expanded regions (at spatial resolution of 70 μm) that reveal excellent correlation with the anatomical features of H&E-stained tissue images. Moreover, to emphasize the importance of imaging at higher spatial resolutions (at pixel size of 25 μm), the smaller bile ducts located in the proximity of larger blood vessels are presented in Figure 4d, e. In these experiments, the bile acids were detected even in the ducts of small diameters such as 50–100 μm, which demonstrates the great potential of the method for studying human liver samples, including the molecular visualization of interlobular and, with further improvement of lateral resolution, the intralobular bile ducts. Of note, our experiments and results showed a minor tendency to delocalization of bile, during cryosectioning, thaw-mounting, and/or matrix spraying, even though a tightly controlled sample preparation protocol was used (see Experimental). While completely eliminating delocalization from the first two processes can be difficult (if not impossible), solvent-free matrix deposition such as dry-coating or sublimation can avoid delocalization from matrix spraying. Such an approach will be crucial in the next stage of our work, where we investigate images of smaller features of the biliary network.

### Proton-Bound Dimers of Bile Acids and Taurine

**Figure 5:**
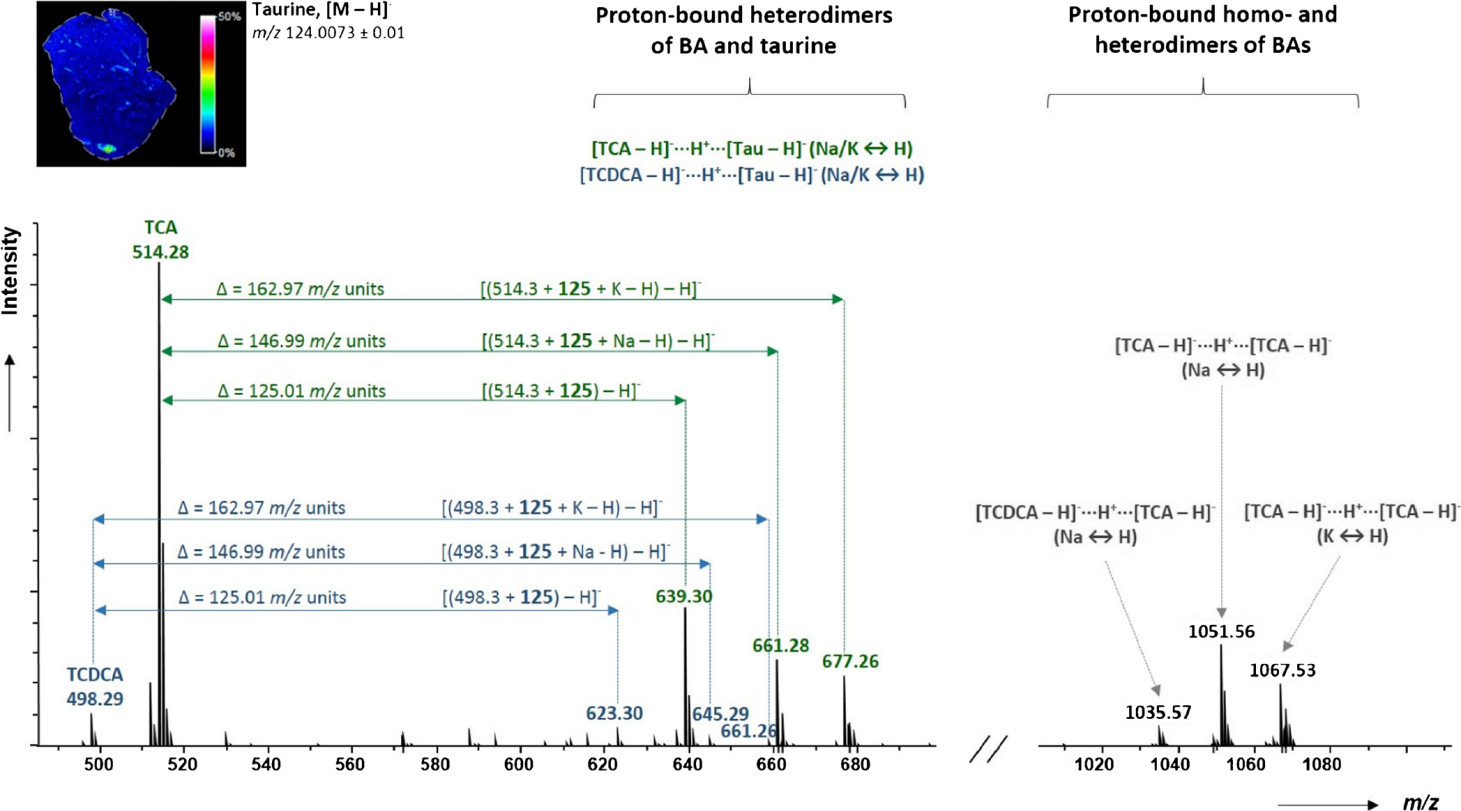
Single MSI pixel mass spectrum from the bile duct region in the range *m/z* 490–1100, showing signals from proton-bound dimers formed between bile acids (homo- and heterodimers) as well as between bile acids and taurine. The 2D ion image on the upper left illustrates the distribution of taurine in the liver section

Several recent LC-MS/MS studies have reported the identification of a wide range of bile acids in liver and bile samples [33, 34, 62]. We interrogated our MSI data sets for these and additional molecules, which are associated with the gall bladder and bile ducts regions. In addition to the manual investigation of selected regions of interest (ROI), we also applied mass defect filtering (MDF) based on average mass spectra from the MSI experiments, to identify further potential metabolites of bile acids. For this purpose, we chose an unconjugated monohydroxylated bile acid, litocholic acid (LCA), as core substructure and set a mass defect filter window of ±50 mDa around the exact mass of LCA (to include the most common bile acids conjugations) over a broader mass region from *m/z* 400 to 700. This combined approach of data processing revealed >20 promising monoisotopic *m/z* value candidates. Most notably, two interesting groups of signals with the highest relative signal intensities were found in the ranges between *m/z* 620 and 680 and from *m/z* 1,020 to 1080, which are shown as single MSI pixel mass spectrum in Figure 5. Surprisingly, searching common databases (LIPID MAPS and METLIN) did not give any positive identifications. Moreover, the measured accurate mass shifts from the chosen core structure did not match any known or previously observed biotransformations. As bile acids have been reported to form proton-bound (mixed) dimers during electrospray ionization [50, 51] as well as solvent adducts in positive ion mode and carboxylic acid adducts in negative ion mode [63], we also investigated the observed mass shifts for potential formations of these cluster ions.

As a result, we were able to identify two species of proton-bound dimers formed during the MALDI-MSI experiments (Figure 5). Firstly, cluster ions of the general structure [BA-H]^-^···H^+^···[BA-H]^-^, which are dimers of TCA/TMCA (*m/z* 1051.56 and 1067.53), and a mixed dimer of TCDCA and TCA/TMCA (*m/z* 1035.57). Secondly, [BA-H]^-^··· H^+^···[Ta-H]^-^ adducts, which were, in fact, mixed dimers of TCA/TMCA and taurine (*m/z* 639.30, 661.28 and 677.26) or TCDCA and taurine (*m/z* 623.30, 645.29 and 661.26). Importantly, the high abundance of taurine across the examined liver sections is readily seen in the ion image of *m/z* 124.0073 ± 0.01 (from the same tissue section), which corresponds to deprotonated taurine, providing additional corroboration of our findings on the identified bile acids and the taurine dimer formation. Of note, all identified dimers showed the same localization and spatial distributions as the deprotonated BAs (Figure S3, Supplementary Information).

To unambiguously confirm the results on the PBD, we examined an equimolar mixture of standard solutions of TCA, taurine, and potassium chloride by MALDI-FTICR (via 9-AA matrix and dried-droplet sample preparation). The results (Figure S4, Supplementary Information) confirmed the previous data on the proton-bound dimers of TCA and taurine. We also performed on-tissue CID experiments of the mass selected cluster ions, by co-adding 16 individual scans of each enhance signal-to-noise ratios, and comparison with CID experiments of standard mixtures. The CID spectra illustrated in Figure S5 (Supplementary Information) showed a single product at *m/z* 498.3 for clusters containing TCDCA (*m/z* 623.3 and 645.3); or at *m/z* 514.3 for dimers containing TCA/TMCA (*m/z* 639.3, 661.3, 677.3 and 1051.6). Interestingly, MS/MS spectra of mixed dimers of bile acid and taurine did not show the product ions related to taurine, which can readily be explained by high gas-phase acidity of taurine-conjugated bile acids [51].

### Implications of PBD for MALDI-MSI: Impact on [M – H]^-^ Intensities

In addition to considerably complicating mass spectral interpretation, the formation of proton-bound dimers also diminishes signal intensities of the desired [M – H]^-^ peaks. This effect becomes even more detrimental in MALDI-MSI experiments, in particular at high lateral resolutions, where the amount of analyte in a single pixel tissue area is very limited and often requires operating at or close to the detection limits of the method. Interestingly, the intensity ratio of PBD to deprotonated BA (PBD/BA) measured in our imaging experiments varied across the regions of the tissue section (average mass spectra and PBD/BA values for four regions are shown in Table S1 and Figure S6, Supplementary Information). Of note, the heterodimers of TCA/TMCA and taurine contributed most significantly to the overall sum intensity of all PBDs, and the highest total PBD/BA ratios were observed for large and small bile ducts (0.64 and 0.78, respectively). This suggests a dependence of PBD formation on the tissue concentration of the contributing molecules and the ion density after MALDI, respectively, which will vary across the tissue.

**Figure 6:**
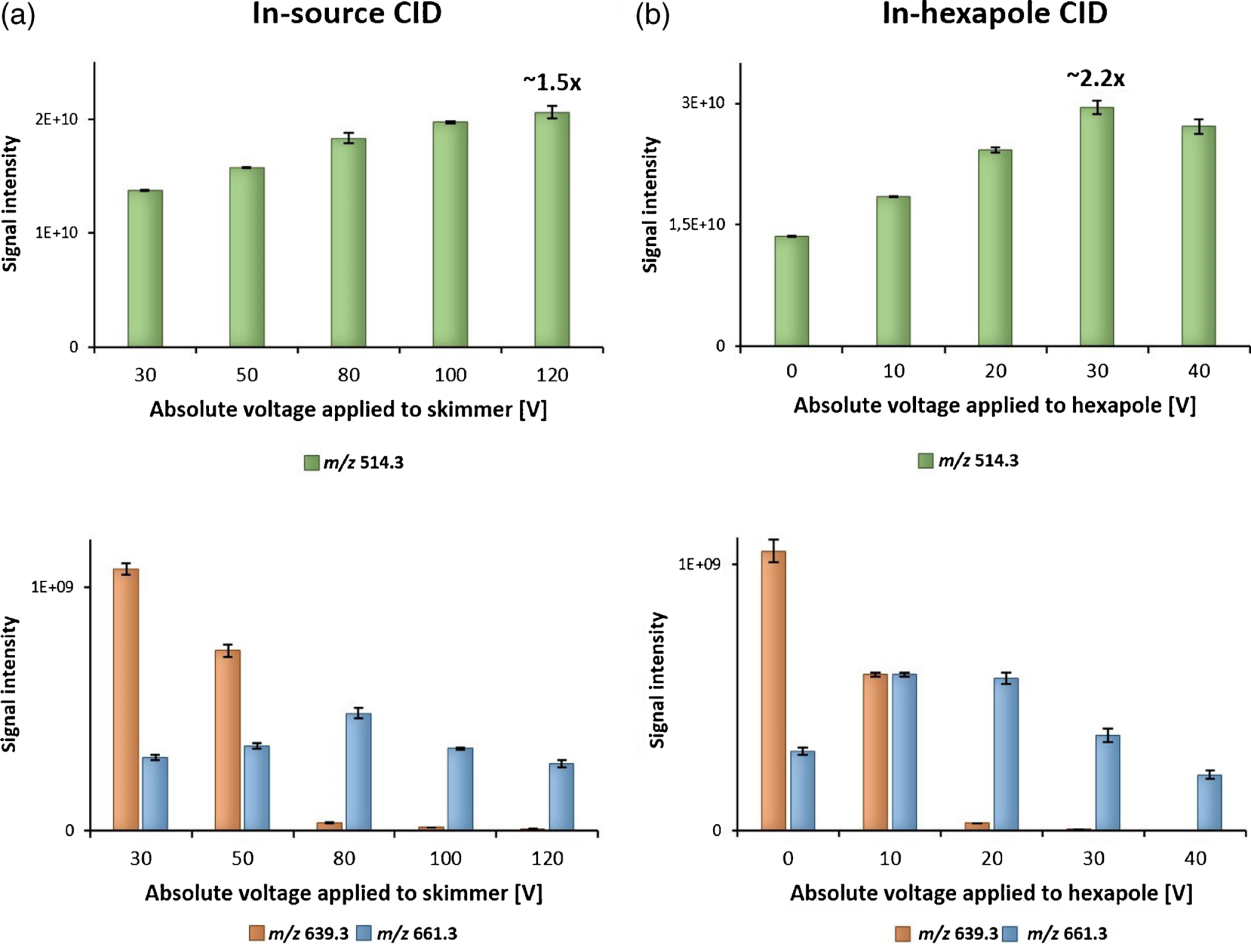
Bar chart showing signal intensities for three different *m/z* values, *m/z* 514.3 (deprotonated TCA), *m/z* 639.3 (mixed dimer of TCA and taurine), and *m/z* 661.3 (sodiated dimer of TCA and taurine) obtained by direct infusion ESI-FTICR and two different approaches for CID: in-source CID (increasing voltage applied to skimmer) **(a)** and in-hexapole CID **(b)**

Here, we introduce a simple technique for use on mass spectrometry platforms with a quadrupole collision cell or ability for efficient in-source CID prior to FTICR mass analysis. Essentially, PBD are dissociated, resulting in increased signal intensities for the deprotonated molecule. We implemented two different approaches for CID in non-selective broadband mode: (1) dissociation in the transport region between skimmer and ion funnel, often referred to as “nozzle-skimmer dissociation” or “in-source CID”; (2) dissociation in the hexapole collision cell, while maintaining full transmission of the entire *m/z* range of interest, without any prior precursor ion isolation. To demonstrate the feasibility of the two CID methods, the standard mixture of TCA and taurine was examined using direct infusion ESI-FTICR. The ESI source was deliberately chosen here, as it allowed delivery of a constant ion current, while MALDI would have been strongly affected by crystallization irregularities. As seen in Figure 6, increasing either the absolute voltage applied to the skimmer or the collision energy for the hexapole cell resulted in an increase of signal intensities for [TCA – H]^-^ ion (*m/z* 514.3), while intensities for both dimer signals (*m/z* 639.3 and 661.3) decreased. Of the two approaches, broadband CID in the hexapole was slightly more efficient, providing an increase of factor ~2 of the deprotonated TCA signal. Interestingly, the sodiated form of the mixed dimer of TCA and taurine (*m/z* 661.3) exhibited higher stability compared with the ‘pure’ PBD (*m/z* 639.3). This is, of course, often observed with sodiated species undergoing CID versus protonated species of the same molecule; for PBD, it can be explained by different gas-phase conformations of the cluster ions, where the sodium cation contributes to the bonding, creating, in fact, a sodium-bound dimer [64]. Of note, some other structural factors may also play a role in the stability of the gas-phase clusters, including formation of multiple inter- and intramolecular hydrogen bonds [50, 65], as well as different positions of the bonding hydroxyl groups at the ring [66, 67].

Furthermore, to demonstrate the utility of the developed method for MSI, a model MALDI imaging experiment was designed in which an equimolar mixture of TCA and taurine was homogenously sprayed across the liver section placed on a glass slide, followed by deposition of 9-AA matrix. Two regions (glass slide and liver tissue) were then selected for imaging experiments, with and without applying the ‘in-hexapole’ dissociation method (each region was composed of at least 200 single pixels at 70 μm spatial resolution, from which the average mass spectra and PBD/BA values are shown in Figures S7a, b, and 7d, e, Supplementary Material). Applying collision energy (30 V) resulted in an increase of the average signal intensity for [TCA – H]^-^ (*m/z* 514.3) by factors of 2.7 and 1.6 for glass and tissue regions, respectively, while the sum of averaged intensities from three heterodimer signals (*m/z* 639.3, 661.3 and 667.3) decreased. Increasing the laser energy might also be considered to be a potentially useful alternative for dimer dissociation. As shown by Dreisewerd and coworkers, however, optimal MALDI-MS performance (=maximum analyte signal intensity at the lowest threshold laser fluence) can be achieved by utilizing a laser energy approximately 2–3 times higher than the ion detection threshold [68], which was also used in the present study. Increasing the laser energy above this value can result in strong ablation from the tissue surface and may lead to increased thermal degradation of the analytes, leading to overall analyte signal deterioration. This was confirmed in our experiments, as shown in Figure S7c, f (Supplementary Material). Therefore, we consider the ‘in-hexapole’ broadband dissociation approach developed here as a superior and gentler means of increasing signal intensities from deprotonated bile acids during MALDI-MSI.

## Conclusions

The primary goal of this study was the visualization of bile acid distributions across mouse livers sections on a molecular level. To our knowledge, this is the first report on the detection and identification of taurine-conjugated bile acids directly from mouse liver sections. The acquired MALDI-MSI data corresponded well with the micro-anatomical features of the mouse liver biliary tract obtained from histological examinations. Furthermore, we have demonstrated the potential of the method for high spatial resolution MALDI imaging at pixel size of 25 μm, which even allows the differentiation of small biliary ducts from blood vessels and liver parenchymal tissue. In addition to MALDI-MSI, our research revealed extensive proton-bound dimer formation between endogenous taurine and taurine-conjugated bile acids. Since formation of these dimers negatively influenced signal intensities of the desired [M – H]^-^ species, a simple method of broadband dissociation was proposed that provided increased signals of the deprotonated species. This deliberate PBD dissociation will be implemented in our future MALDI-MSI studies, in particular in applications where the sensitivity of the method will be the limiting factor; for example, when even higher spatial resolutions than presented here are required (e.g., molecular mapping of small interlobular bile ducts of diameter of ~10 μm for studying cholestasis models [69]), or for bile acid distributions in the brain at much lower concentration levels than in the liver, where they play important signaling roles in neurological disease [70–72]. Other work currently in progress includes a detailed experimental investigation of the exact structural factors stabilizing the different proton/cation-bound dimers of bile acids and amino acids.

## Electronic supplementary material

ESM 1 (DOCX 2.64 mb)
