# Supplementary material for: MALDI Mass Spectral Imaging of Bile Acids Observed as Deprotonated Molecules and Proton-Bound Dimers from Mouse Liver Sections
Source: J Am Soc Mass Spectrom. 2018 Feb 7;29(4):711–22. doi: 10.1007/s13361-017-1886-6 (PMC5889423; doi:10.1007/s13361-017-1886-6)

*Supplementary Material*

**MALDI Mass Spectral Imaging of Bile Acids Observed as Deprotonated Molecules and Proton-Bound Dimers from Mouse Liver Sections**

## Ignacy Rzagalinski^1^, Nadine Hainz^2^, Carola Meier^2^, Thomas Tschernig^2^, Dietrich A. Volmer^1,3^*

*^1^Institute of Bioanalytical Chemistry, Saarland University, 66123 Saarbrücken, Germany*

*^2^Institute of Anatomy and Cell Biology, Saarland University, 66421 Homburg, Germany*

*^3^Department of Chemistry, Humboldt University of Berlin, 12489 Berlin, Germany*

*To whom correspondence should be addressed:

Prof. Dr. Dietrich A. Volmer

Department of Chemistry

Humboldt University of Berlin

Brook-Taylor-Str. 2

12489 Berlin, Germany

Tel: +49 30 2093 7575

Email: dietrich.volmer@hu-berlin.de


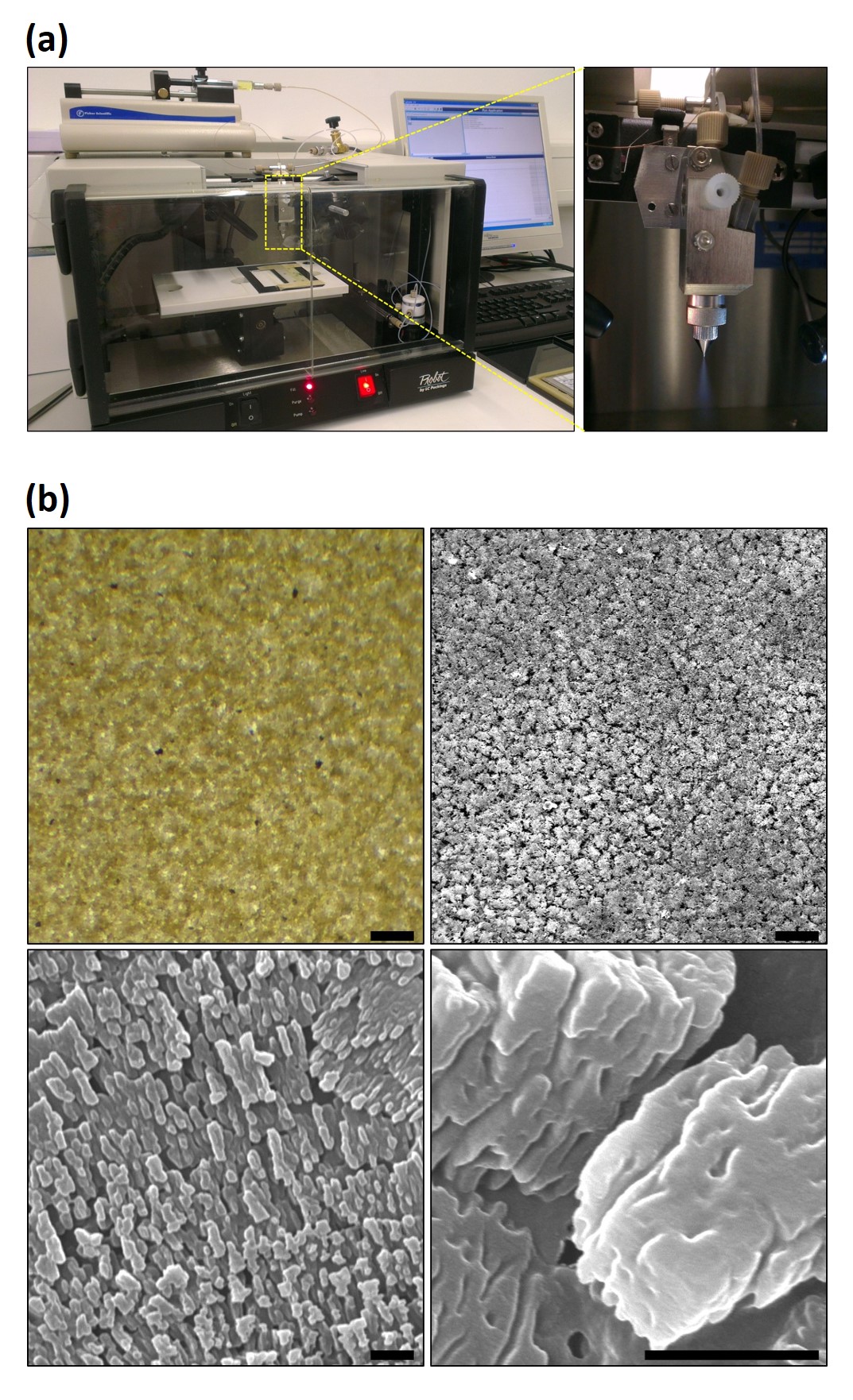


**Figure S1.** **(a)** Home-built robotic sprayer based on the Probot micro fraction collector platform, syringe pump and micro spraying nozzle, providing a fine and highly reproducible spray. **(b)** Images of 9-AA matrix sprayed onto the surface of the control liver section: light microscope image at 5× magnification (top left) and SEM images at 50× magnification (top right), 5,000× (bottom left) and 20,000× (bottom right). Scale bars: 100 µm (top left and right) and 1 µm (bottom left and right).


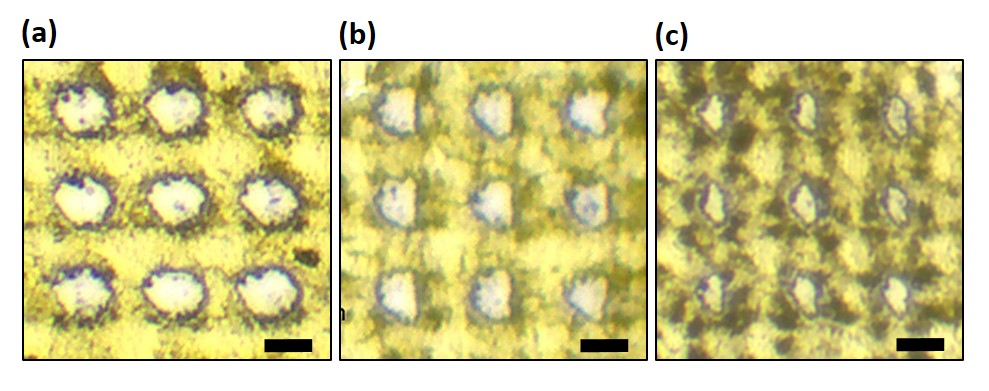


**Figure S2.** Light microscope images of the ablated areas of 9-AA matrix deposited onto the mouse liver section and irradiated with different number of laser shots per pixel: 1000 **(a)**, 500 **(b)** and 200 **(c)**. Other laser settings were: minimum laser beam focus setting (smallest option on commercial Bruker solariX 7T instrument); laser power, 10%; repetition rate, 1 kHz. Scale bar: 50 µm.


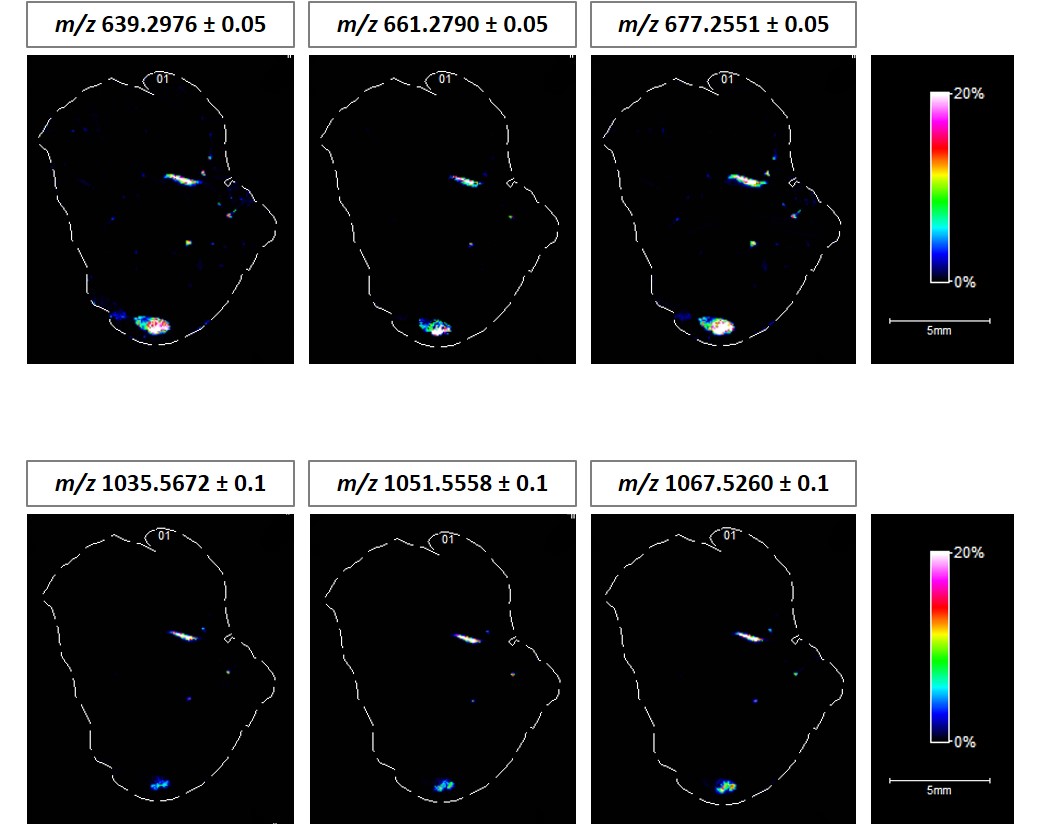


**Figure S3.** MS ion images representing spatial distributions of the identified TCA/TMCA-containing proton-bound dimers at *m/z* 639.2976 ± 0.05, *m/z* 661.2790 ± 0.05, *m/z* 677.2556 ± 0.05, *m/z* 1035.5672 ± 0.05, *m/z* 1051.5558 ± 0.05, and *m/z* 1067.5260 ± 0.05 across the whole section of mouse liver (pixel size, 70 µm). All ion images were normalized to the 9-AA matrix signal.


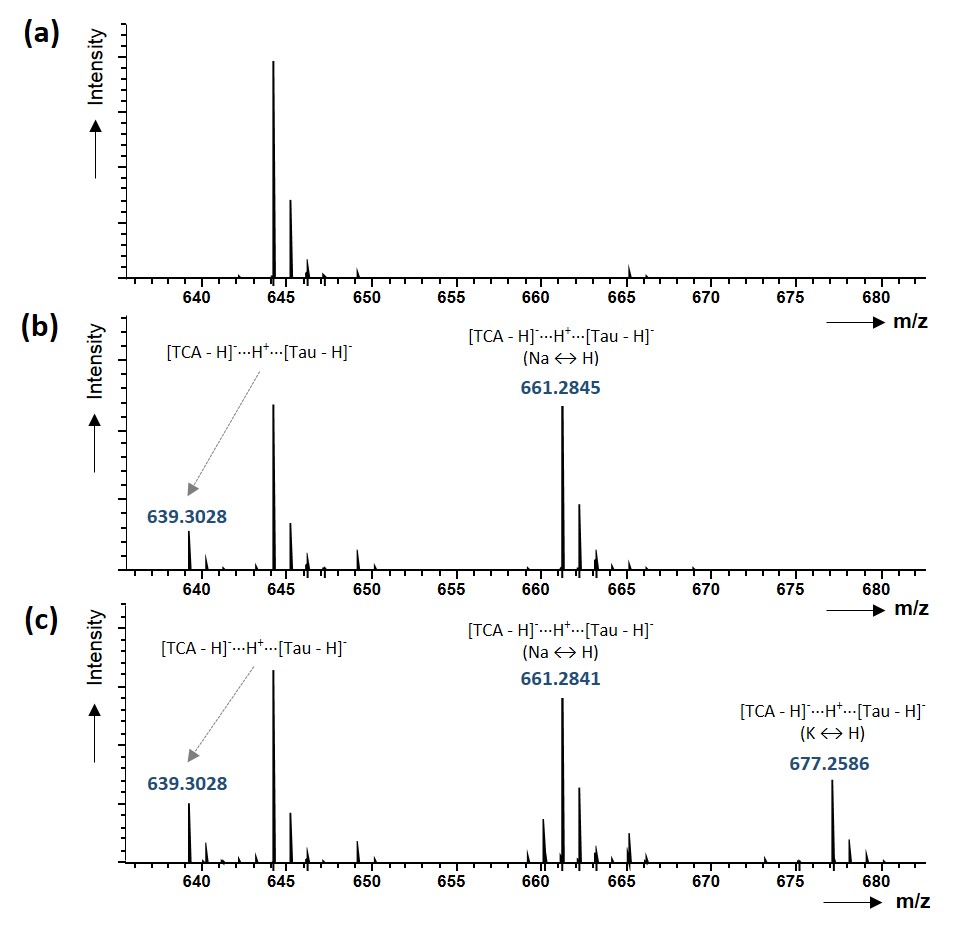


**Figure S4.** MALDI-FTICR mass spectra acquired after dried-droplet sample preparation using 9-AA matrix + standard mixtures of TCA **(a)**, TCA + taurine **(b)** and TCA + taurine + KCl **(c)**.


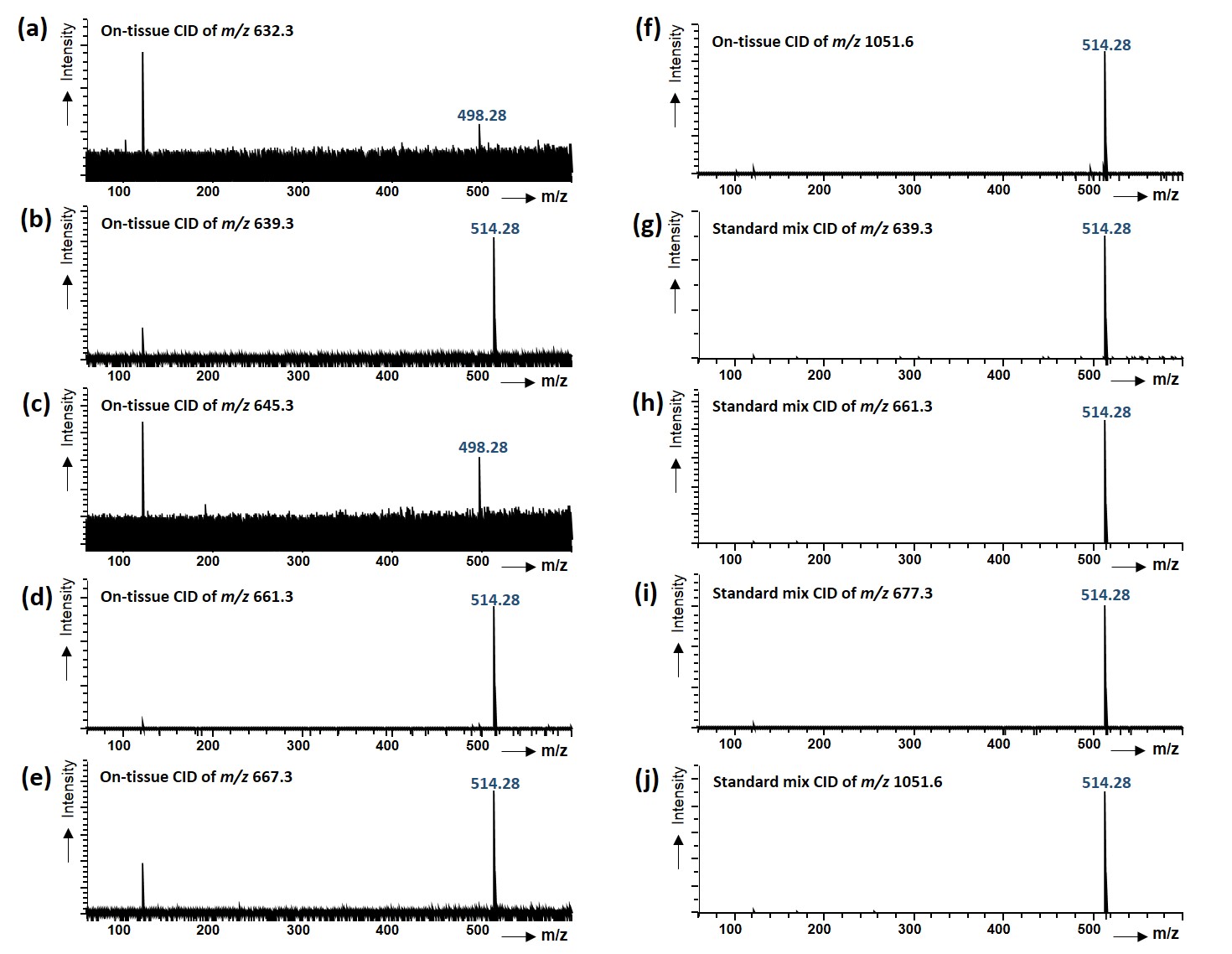


\

**Figure S5.** CID experiments of *m/z* selected dimers obtained directly from mouse liver tissue sections **(a-f)** or from standard mixture of TCA, taurine and potassium chloride **(g-j)**. (Note: *m/z* 123 is an artifact of the FTICR instrument used in the study.)


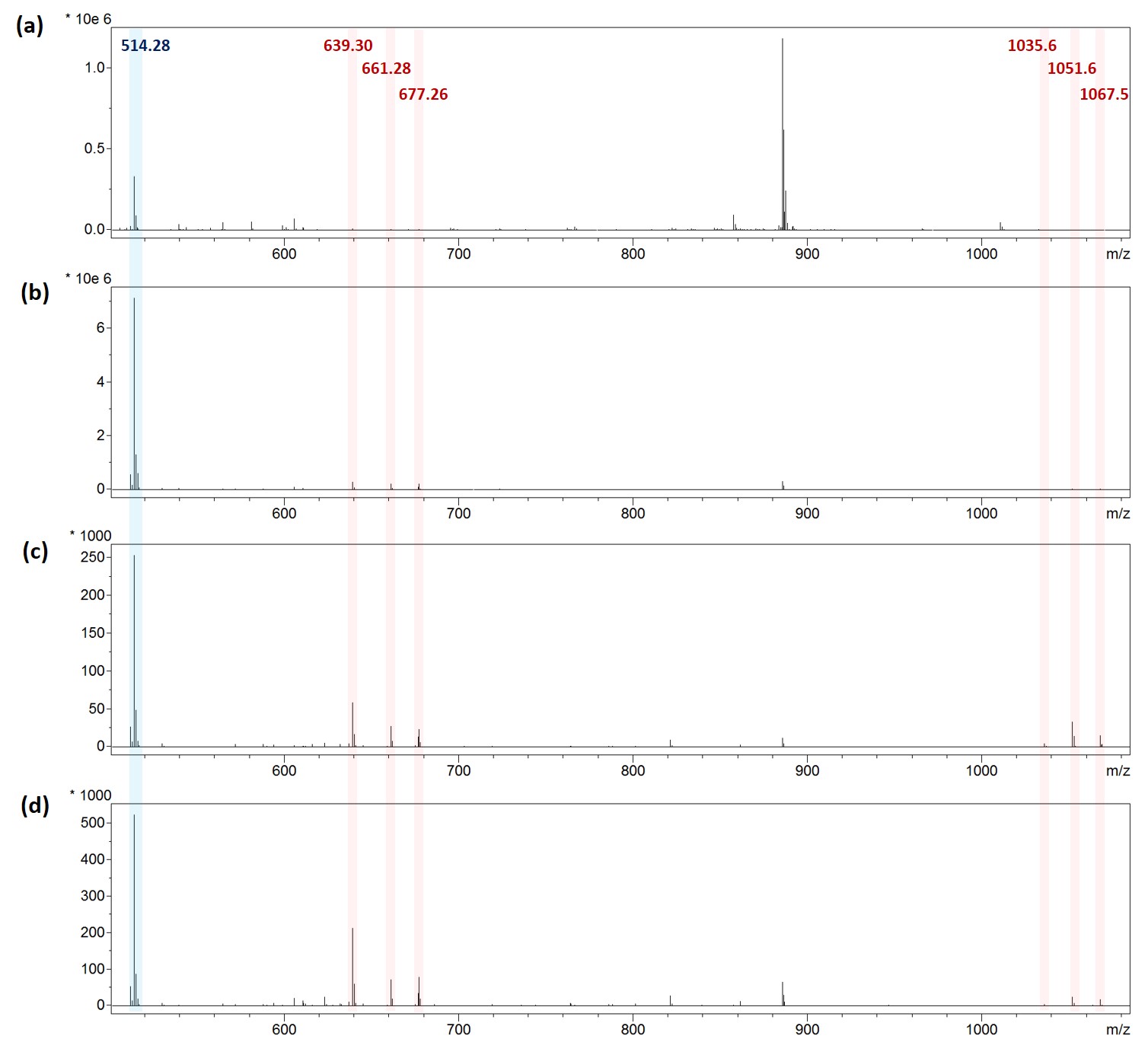


**Figure S6.** Averaged mass spectra from different selected regions of interest (ROI): whole liver tissue section (22,136 single mass spectra averaged) **(a)**, gall bladder (125 single mass spectra averaged) **(b)**, the large bile duct of which the MS image is shown in Figure 4c (29 single mass spectra **(c)**, and the small bile duct of which the MS image is shown in Figure 4e (4 single mass spectra averaged) **(d)**. All mass spectra were obtained from MS imaging experiments performed at 70 µm spatial resolution.


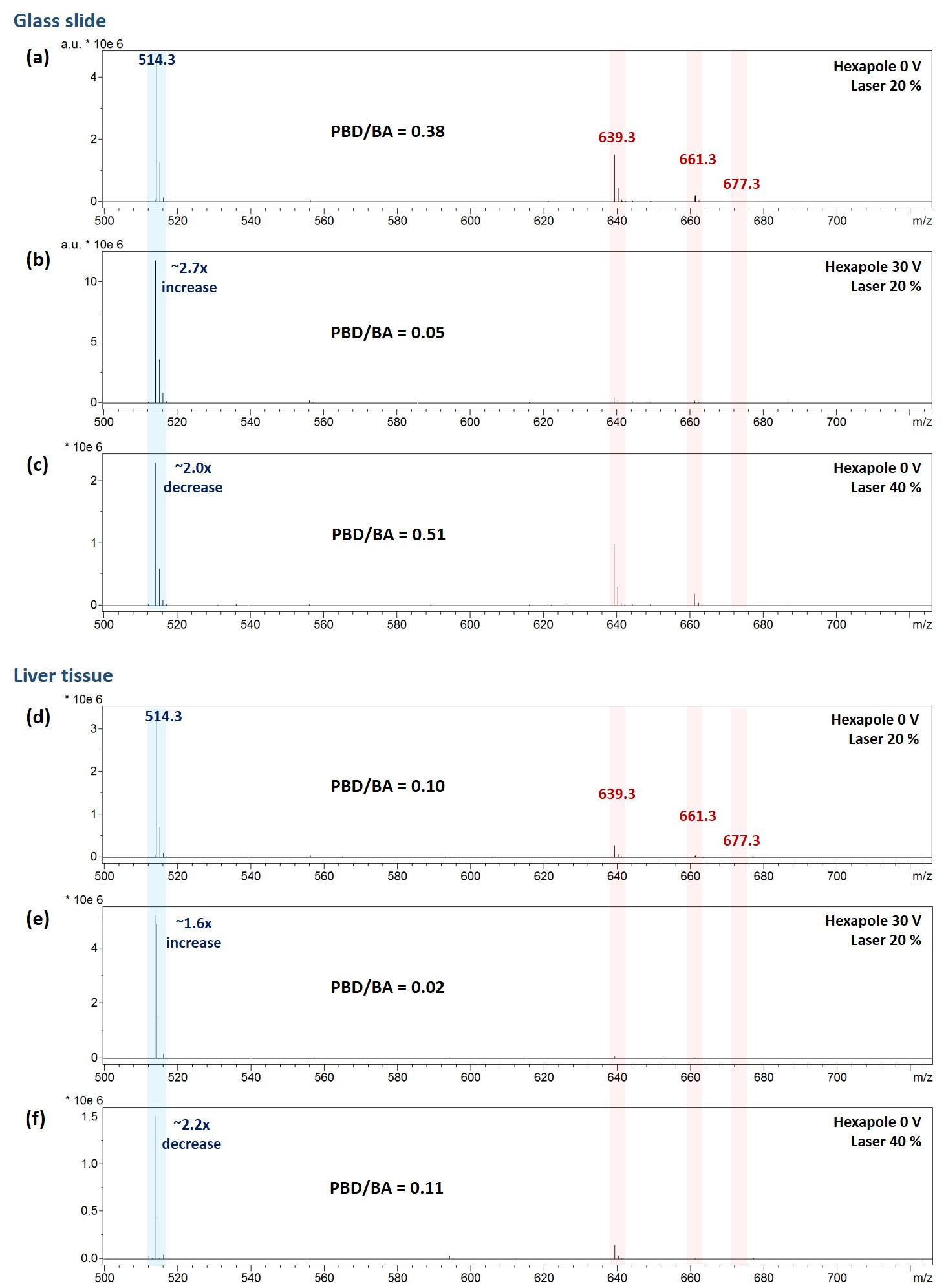


**>>a**

**Figure S7.** Average mass spectra from model MALDI imaging experiments conducted on glass slide **(a-c)** and liver tissue **(d-f)** regions sprayed homogenously with an equimolar mixture of TCA and taurine and imaged using three different instrumental settings: no collision voltage at hexapole (0 V) and laser energy set to optimum conditions (20%) **(a, d)**; 30 V at hexapole and laser power at 20 % **(b, e)**; 0 V at hexapole and doubled laser power (40 %) **(c, f)**. All average mass spectra were obtained from at least 200 single points (pixel size, 70 µm). The PBD/BA values were calculated based on the average sum intensity of all heterodimers (TCA/taurine) and average intensity of deprotonated TCA.

**Table S1.** Ratios of average signal intensities of different TCA/TMCA-containing proton-bound dimers and the average signal intensity of deprotonated TCA/TMCA. The values were obtained from four different regions of interest (ROIs) – whole liver section, gall bladder, large bile duct and small bile duct – by averaging mass spectra from 22136, 125, 29 and 4 single points, respectively (pixel size, 70 µm).


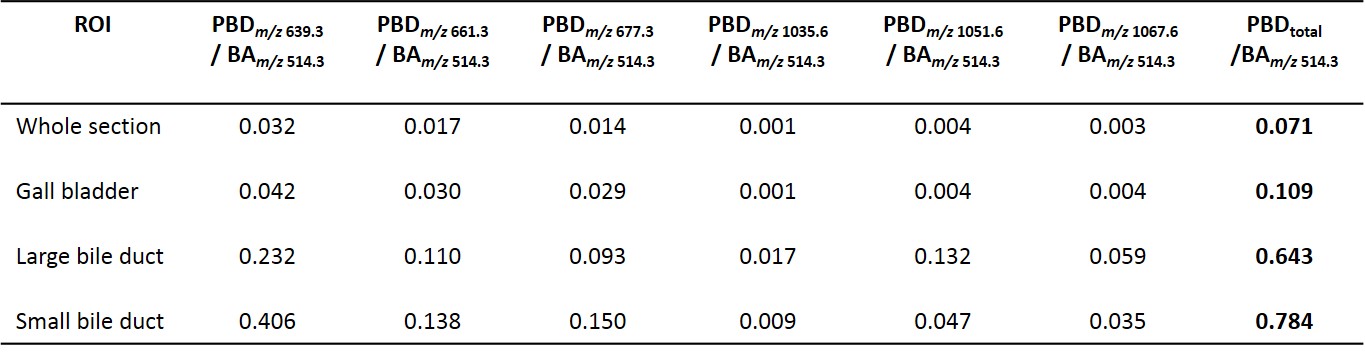

Supplement: Supplementary file 1 — (DOCX 2.64 mb) [file 13361_2017_1886_MOESM1_ESM.docx]
